# An Unusual Cause of Necrotising Fasciitis in a Young Male with Juvenile Dermatomyositis

**DOI:** 10.1155/2022/8758263

**Published:** 2022-08-09

**Authors:** Adelaide Ankomaa Asante, Josephine Nsaful, Dzifa Dey

**Affiliations:** Korle Bu Teaching Hospital, P.O. Box 77, Korle Bu, Accra, Ghana

## Abstract

Juvenile dermatomyositis (JDM) is a rare condition worldwide, affecting children younger than 16 years. It is characterized by weakness in the proximal skeletal muscles and a pathognomonic skin rash. Patients with JDM develop complications that are usually a consequence of vasculopathy affecting multiple organ systems. Occult gastrointestinal (GI) perforation is an uncommon complication and is associated with an increased risk of mortality due to a delay in diagnosis. We report on a 14-year-old male with JDM with an aggressive course over two years and severe clinical manifestations. The patient developed necrotizing fasciitis, an unusual rapidly progressing lethal infection of the fascia resulting from bowel contents seeping from multiple intestinal perforations. This case, less commonly seen in males, highlights the occurrence of multiple phenomena—JDM complicated by skin and gastrointestinal vasculopathy with resultant development of multiple GI perforations and consequently life-threatening necrotizing fasciitis of the leg. Physicians need a high index of suspecting GI perforation in JDM patients as the delayed recognition of this complication can result in significant morbidity and/or mortality since the typical symptoms of perforation may be absent.

## 1. Introduction

JDM belongs to a group of inflammatory muscle diseases known as idiopathic inflammatory myositis (IIM), a systemic inflammatory autoimmune condition that affects primarily the proximal skeletal muscles as well as the skin. It manifests as symmetrical proximal muscle weakness, a reddish-purple rash on the upper eyelids with periorbital edema (heliotrope rash) or erythematous, papulosquamous eruption over the dorsal surfaces of the knuckles (Gottron papules) or flat, and erythematous rash over the dorsal surfaces of the knuckles (Gottron sign). Its occurrence is mainly in children below the age of sixteen years, with an average age of onset of 7 years and a higher female to male preponderance [1].

JDM is described as a vasculopathy based on the pathophysiology of the disease and this is responsible for most of the musculoskeletal and extramuscular manifestations of the disease [1, 2].

In addition to the progressive muscle weakness and characteristic skin rashes, cutaneous ulcerations which are a consequence of occlusion of the dermal vessels may be evident in some patients [1]. Dystrophic calcifications (calcinosis) affecting pressure points such as the knees, elbows, and buttocks may be present at the time of diagnosis; however, in some patients, it presents within the first 3 years of onset of the disease and in others as late as 20 years after the illness has occurred [1]. GI involvement occurs later as the disease progresses and manifests as dysphagia, malabsorption, abdominal pain, rectal bleeding, intestinal ischemia, pneumatosis, bowel obstruction, or perforation [3].

In 2017, the revised classification criteria by EULAR/ACR (European Alliance of Associations for Rheumatology/American College of Rheumatology) for adult and juvenile IIMs were developed,replacing the Bohan and Peter classification [4]. This includes age, four variables related to muscle weakness, three related to skin, variables related to laboratory investigations, and other clinical findings [4]. Each variable is assigned a weighted score and the total score relates to the probability of having IIM (Table 1). Based on these scores, patients can be classified as having definite IIM (a score of ≥7.5 if muscle biopsy result is unavailable or ≥8.7 with muscle biopsy available and a probability ≥90%) and probable IIM (a score of ≥5.5 if muscle biopsy result is unavailable or ≥6.7 with muscle biopsy results and a probability ≥55%) [4]. A score of <5.3 without muscle biopsy results or <6.5 with muscle biopsy and a probability of <50% rules out IIM. Patients with scores probabilities between 50 and 55% are classified as possible IIM [4]. The EULAR/ACR criteria allow a scoring system when muscle biopsy is unavailable and this is particularly useful in the pediatric population where biopsies are not frequently done [5]. A patient classified with IIM by the EULAR/ACR classification criteria (probability of IIM ≥55%) is further subclassified with a classification tree [4].

Glucocorticoids and methotrexate (MTX) remain the first line of treatment of choice for JDM [6]. For refractory disease, other disease-modifying antirheumatic drugs (DMARDs) such as cyclosporin and biologic agents like rituximab can be used [3, 6].

Worldwide, the prevalence of JDM is unknown; however, the incidences in the United States and the United Kingdom are 2.5–4 and 1.9 cases per million population, respectively [7]. In Africa, specifically in the sub-Saharan region, data on the incidence of JDM are limited. Few case reports have been cited in Nigeria and Kenya [8, 9]. Furthermore, there have been sparse reports on the GI manifestations in patients with JDM.

We report a case of a 14-year-old boy, who presented paradoxically with chronic right-sided hip pain with a sequel of necrotizing fasciitis and intestinal perforation due to vasculopathy.

## 2. Case Report

The patient was diagnosed with JDM in 2019 at the age of 12 after presenting with a two-month history of generalized swelling, fever, rash over the face and neck, joint pains, and weakness that made him unable to walk, talk, or feed himself. On examination, he had periorbital swelling and heliotrope rash, Gottron's papules, nail fold infarcts, a V-neck rash, and cervical lymphadenopathy. There were multiple tender joints and synovitis. Power was reduced in the proximal muscle groups bilaterally. Results of labs undertaken initially showed elevated creatinine kinase (CK) of 1643 IU/L, antineutrophil antibody (ANA) of 1 : 160, extractable nuclear antigen (ENA) panel negative for all tested antibodies, and normal C3 and C4. The antibodies tested for on the myositis-specific autoantibodies (MSA) were also negative. Initial treatment consisted of oral doses of prednisolone 30 mg daily with subsequent tapering to 10 mg daily, hydroxychloroquine 200 mg daily, calcium 500 mg daily, omeprazole 20 mg daily, and MTX 7.5 mg weekly.

**Figure 1:**
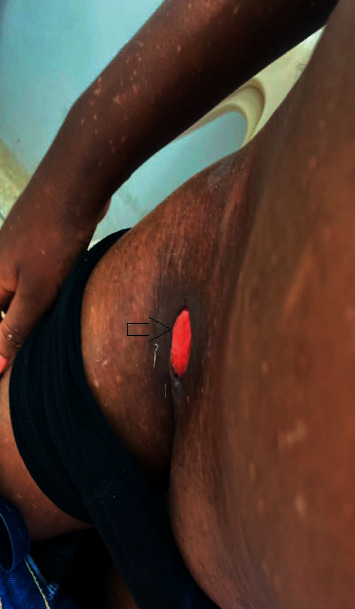
Vasculitic ulcer occurring at the groin. Multiple hypopigmented macules on the anterior abdominal wall, extensor surface of the right upper limb, and thigh.

In a year, although there was mild improvement in the muscle weakness, he developed multiple vasculitic ulcers (Figure 1) which were deep-seated and slow in healing. He received repeated antibiotics due to secondary bacterial infections of the ulcers; thus, MTX was withheld during the periods that he was being treated with the antibiotics.

Eighteen months after diagnosis, the patient complained of persistent right hip pain radiating down the knee and with no response to pain medications. A magnetic resonance imaging (MRI) of the right hip was reported as normal. The pain was managed by administering pulses of intravenous (IV) methylprednisolone 500 mg daily for 3 days after which he was continued on oral prednisolone 30 mg daily and subsequently reduced to 10 mg daily. Due to persistent bacterial infection of the ulcers, MTX was withheld and antibiotic therapy started. He later developed calcinosis which was complicated by contractures of the knee and elbow joints.

The patient was admitted on account of worsening right hip pain three months after his initial complaints. Examination findings revealed swollen knees bilaterally, coupled with tenderness and differential warmth. New areas of calcinosis were observed. Although tachycardic, the patient's temperature remained normal. CK level was 34 IU/L, blood cultures yielded no bacterial growth, and MRI and ultrasound of the hip ruled out septic arthritis or avascular necrosis of the right hip. Pulse therapy with 500 mg of IV methylprednisolone was administered for 3 days and later switched to oral prednisolone 30 mg daily.

**Figure 2:**
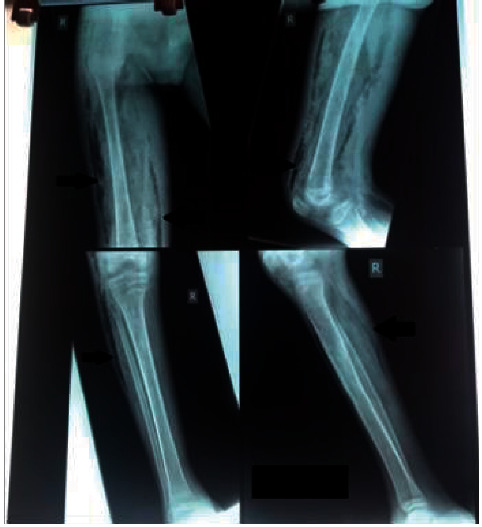
X-ray images showing gas pockets in the right thigh and leg.

During admission, the patient complained of severe pain in the right knee, thigh, hip, and gluteal area, and by day 10, the right knee was erythematous and had crepitus from the midthigh to the distal 1/3rd of the right tibia with associated gross swelling of the entire right thigh. He was tachycardic and tachypnoeic and had gaseous distension of the abdomen although bowel sounds were present. The patient did not vomit during this period and was passing stools normally. An ultrasound scan of the abdomen and right leg were done on suspicion of necrotizing fasciitis which revealed extensive edema and gas pockets within the subcutaneous tissues of the right leg, consistent with necrotizing fasciitis. The abdominopelvic ultrasound scan was, however, normal. An X-ray of the affected limb was also suggestive of necrotizing fasciitis (Figure 2). Intravenous meropenem 500 mg and vancomycin 500 mg both given at 8 hourly intervals were commenced and the patient was prepared for debridement.

**Figure 3:**
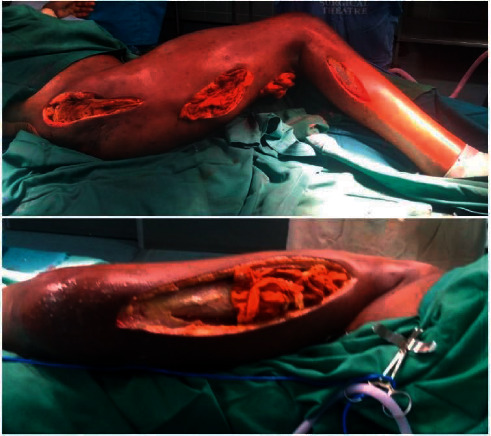
Initial debridement-offensive abscesses in the anterior and lateral aspects of the right thigh, right hip, and right lower leg.

**Figure 4:**
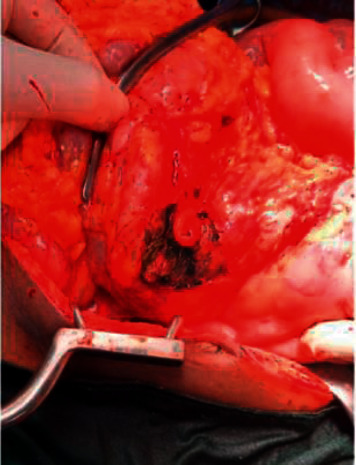
Areas of perforation on the proximal ascending colon.

Intraoperatively, extensive necrotizing fasciitis involving the anterior and lateral aspects of the thigh, leg, hip, and posterior aspects of the thigh and hip with pockets of copious offensive abscesses were found (Figure 3). The abscesses were drained, while the necrotic fascia was debrided adequately and irrigated. A vacuum-assisted wound closure device was placed over the right thigh and leg. Gram staining and culture of the abscess yielded *E. coli* sensitive meropenem which he was already on. Three days after the debridement, it was observed that the dressing around the right thigh was stained with feculent matter. A methylene blue dye test was conducted which confirmed the presence of an enterocutaneous fistula. Exploratory laparotomy was done during which three posteriorly located perforations (Figure 4) in the proximal ascending colon, communicating with the gluteal wound, were found. There was no soiling of the peritoneum. Limited right hemicolectomy and an end ileostomy were carried out. Histopathology of the resected ascending colon revealed transmural acute on chronic inflammatory changes in areas with full-thickness necrosis.

After surgery, the patient developed a surgical site infection and suffered electrolyte imbalances (hypokalemia, hypomagnesemia, and hypocalcemia) which did not respond to replacement therapy. He passed away 36 days after the surgery.

## 3. Discussion

The above case represents a young boy who had severe JDM resulting in bowel perforation and necrotizing fasciitis of the right lower limb.

JDM is the most common inflammatory myopathy of childhood and a rare systemic autoimmune vasculopathy [1].

**Figure 5:**
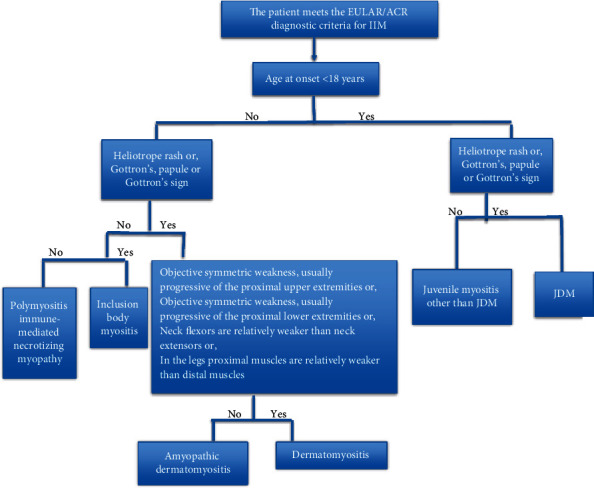
Classification tree for subgroups of IIM [9].

The diagnosis of JDM is rarely made in children living in sub-Saharan Africa. Aliu et al. assigned this partly to limited availability of diagnostic tools and partly to the limitations with the use of the Bohan and Peter criteria [9]. In Kenya, a definite diagnosis could not be made in a patient suspected of having JDM due to the inability to perform muscle biopsy or electromyography studies which are needed in the Bohan and Peter criteria [8]. One advantage of the EULAR/ACR classification criteria (Table 1) is that IIMs can be accurately classified without muscle biopsy data [4]. Based on this, our patient was classified as having definite IIM as shown by the presence of muscle weakness, skin manifestations, and elevated serum levels of CK/LDH, giving him a score of 14.1 and a probability of 90%. These new classification criteria are useful in our subregion where electromyography and biopsies are infrequently carried out. The patient was further subclassified as JDM using the classification tree (Figure 5).

GI manifestations of JDM such as ulceration and perforation are features of severe disease and are associated with a poor prognosis [10]. These complications make up 22–37% of JDM [1]. Compared to adult-onset DM, GI events are more common in JDM [11]. Mortality associated with GI complications may be as high as 38;% [12]. Patients with extensive cutaneous manifestations are more likely to develop GI symptoms [5]. Our patient also had widespread skin involvement and later developed GI perforation. Occlusive vasculopathy is an important factor in the development of cutaneous ulceration, intestinal ischemia, and finally intestinal perforation [13].

It is recommended that MTX is withheld in patients with infections until the antibiotic course has been completed and the clinical symptoms have resolved [14]. However, treatment with MTX was interrupted in this patient due to recurrent infections. In South Africa, it has been reported that patients with calcinosis were at higher risk of developing *Staphylococcus aureus* infections; hence, these patients were put on prophylactic cloxacillin [15]. Our patient was, however, not on prophylactic antibiotic therapy. Calcinosis is a complication that develops a few years after diagnosis and is associated with delayed or inadequate treatment. With this patient, it was difficult to control disease activity as MTX had to be withheld at a point and this could have contributed to the development and progression of calcinosis. Although treatment strategies have been proposed to target calcinosis in JDM, none of these have been accepted as standard therapy. The approach, therefore, is to treat the underlying inflammatory condition until the disease is inactive and allow the body to resorb the calcium on its own [5, 16].

Although the clinical and diagnostic significance of the MSA remains unclear and requires further research, Xu et al. discovered that all five patients who had developed GI perforation were positive for the anti-NXP2 antibody [12]. Likewise, a retrospective study of JDM patients who developed GI complications found that anti-NXP2 and TIF1-gamma antibodies were present on the MSA panel [10]. However, all the antibodies on the MSA panel for this patient were negative. It is worthy to mention that the MSA panel did not include these two antibodies; therefore, we cannot tell whether their presence may have played a role in the patient's disease course.

Intestinal vasculitis along with chronic steroid use predisposes patients with JDM to developing ulcerations and perforations [17]. Perforations are likely to happen as part of the natural history of the disease; however, this process may be accelerated by the long-term steroids by inhibiting the inflammatory response and hindering the normal processes of repair and mucosal regeneration [10, 17, 18]. Atrophy of lymphoid elements associated with the use of steroids causes thinning of the bowel wall, thus compromising the integrity of the gut wall and rendering it easy to perforate [17]. Warshaw et al. reported the occurrence of colon perforation in a patient with systemic lupus erythematosus (SLE) who had evidence of vasculitis and had been on steroid therapy as well [17]. Similar findings have been corroborated in other case reports where GI events occur shortly after pulses of methylprednisolone had been administered [3, 10, 18]. A possible causal relationship between high-dose steroids and the development of intestinal perforation has been proposed based on this [10]. Likewise, the patient had been on relatively high doses of steroids and had received pulses of methylprednisolone during admission, and these could have also accelerated the development of perforations in addition to the ongoing GI vasculopathy. The immunosuppressive and anti-inflammatory actions of the high doses of steroids administered could have contributed to the rapid progression and further worsening of the infection.

GI tract involvement has been linked to inflammatory vasculopathy which may be acute or chronic. According to Mamyrova et al., a histopathology report on two patients known to have JDM and later diagnosed with GI perforation showed evidence of chronic end-arteropathy typified by narrowing and complete occlusion of small and medium vessels [18]. The patient had transmural acute chronic inflammatory changes. Acute vasculitis results in arterial and venous intimal hyperplasia and subsequent occlusion of vessels by fibrin thrombi in the mucosa, submucosa, and serosal layers of the bowel. This narrowing and occlusion of small and medium-sized arteries leads to ischemia [11]. Acute vasculitis is associated with poorly controlled disease [10, 11]. Although CK levels were normal during admission, the patient had clinically active disease. Unfortunately, disease activity was not objectively assessed using tools such as the Pediatric Rheumatology International Trials Organisation (PRINTO)/EULAR/ACR core set of clinical measures for the assessment of response to therapy in patients with JDM. However, at the time of admission, he was not on full treatment for JDM as the MTX had been withheld months earlier due to recurrent secondary bacterial infections of the vasculitic ulcers. Chronic enteropathy, which may also arise from long-term therapy with steroids, also plays a role in the pathogenesis of ischemic ulceration [18].

Patients typically present with persistent abdominal pain [1, 13]. Early identification of the perforation can sometimes be daunting, with many missing this life-threatening complication. Perforations may be diagnosed as late as 1 month after the initial presentation of abdominal pain [12]. Interestingly, this young boy did not have any symptoms of perforation such as abdominal pain or vomiting. In a case report by Xu et al., three patients with JDM presented with abdominal pain which initially improved after administration of high-dose steroids and immunosuppressant therapy. However, these symptoms recurred after a few weeks, and a diagnosis of GI perforation was made after further investigations [12]. Waiting for radiological evidence of perforation may also delay the diagnosis as repeated scans were needed to detect the perforation in some instances [12, 18]. A single normal radiograph in a patient with persistent abdominal pain and JDM does not exclude the likelihood of perforation, and hence, multiple imaging is required [18]. Earlier publications reported GI perforations occurred averagely between 4 and 13 months from the diagnosis of JDM [18]. However, Mamyrova et al. documented 2 cases in which perforation occurred at 16 and 25 months [18]. Comparably, this patient developed GI complications 18 months after the first diagnosis. Therefore, physicians must develop a high index of suspicion for perforation in patients who have persistent abdominal pain after temporary relief from the treatment.

Necrotizing fasciitis is a destructive soft tissue infection, and it is a relatively rare condition. This can be classified microbiologically into type 1 and type 2 based on the organism isolated. Type 1 is associated with immunocompromised patients and is caused by both aerobic and anaerobic organisms and is associated with crepitus indicating the presence of a gas-forming organism [19]. On the other hand, type 2 occurs in healthy individuals with no preceding history of trauma. The commonly implicated organisms for type 2 are *Streptococcus pyogenes* and *Staphylococcus aureus* [19]. Since this boy was immunocompromised from the long-term steroid therapy together with the underlying autoimmune condition and the isolation of *E*.*coli*, a gas-forming organism from the abscess in the thigh supports the likelihood of this male having type 1 necrotizing fasciitis.

The abnormal connection between the peritoneal cavity and the right thigh may have contributed to worsening the outcome for the patient. A publication by Bazan et al. described a 16-year-old previously diagnosed and treated JDM but had been in remission for 7 years who presented with a right thigh abscess which resulted from rupture of a nonretrocecal appendix. It was suggested that there was a communication between the peritoneal cavity and the right leg arising from the possibility of changes in the muscle and fascia, as well as fibrosis of the connective tissue, that may have occurred early on from disease activity of the dermatomyositis. These changes may have compromised the integrity of the structures allowing contents to spread down the soft tissue plane [20]. On the contrary, our patient had significant disease activity at the time of perforation which may have accounted for the severity and extent of infection due to the above mechanism described.

In well-resourced countries, children with vasculopathy presenting with life-threatening complications respond well after prompt plasma exchange and systemic corticosteroids together with rituximab [3]. Unfortunately, in resource-limited settings like this patient's, these treatments could not be administered and compounded by the setting of a severe infection.

## 4. Conclusion

This case represents the occurrence of JDM, a relatively rare conditionwhich is less common in males. Also, there have been very few reported cases in sub-Saharan Africa. Vasculopathy can result in profound complications such as persistent skin ulcers. When vasculopathy occurs in unusual sites such as the gastrointestinal tract, peritonitis and its sequelae of sepsis and necrotizing fasciitis may occur. Physicians attending to JDM patients with cutaneous manifestations need a high index of suspicion for GI vasculitis as patients may not show signs of typical intestinal perforation as highlighted in the case presented. Such patients should be observed closely by careful examination.Where the need arises, repeated radiological imaging of the abdomen may be warranted to enable early recognition of these dire complications to prevent significant morbidity and improve the outcome in such patients and ultimately delay mortality.

## Figures and Tables

**Table 1:** The EULAR/ACR classification criteria for adult and juvenile idiopathic inflammatory myopathies 4.

Variable	Score points	
	Without muscle biopsy	With muscle biopsy
Age of onset		
Age of onset of first symptom assumed to be related to the disease ≥18 years and <40 years	1.3	1.5
Age of onset of first symptom assumed to be related to the disease ≥40 years	2.1	2.2
Muscle weakness		
Objective symmetric weakness, usually progressive, of the proximal upper extremities	0.7	0.7
Objective symmetric weakness, usually progressive, of the proximal lower extremities	0.8	0.5
Neck flexors are relatively weaker than neck extensors	1.9	1.6
In the legs proximal muscles are relatively weaker than distal muscles	0.9	1.2
Skin manifestations		
Heliotrope rash	3.1	3.2
Gottron's papules	2.1	2.7
Gottron's sign	3.3	3.7
Other clinical manifestations		
Dysphagia or esophageal dysmotility	0.7	0.6
Laboratory measurements		
Anti-Jo-1 (antihistidyl-tRNA synthetase) autoantibody present	3.9	3.8
Elevated serum levels of CK or LDH or aspartate aminotransferase (AST) or alanine aminotransferase (ALT)	1.3	1.4
Muscle biopsy features, presence of		
Endomysial infiltration of mononuclear cells surrounding, but not invading, myofibres		1.7
Perimysial and/or perivascular infiltration of mononuclear cells		1.2
Perifascicular atrophy		1.9
Rimmed vacuoles		3.1

Note: table does not show the scores of the patient.
